# Genetic analysis of *daf-18/PTEN* missense mutants for starvation resistance and developmental regulation during *Caenorhabditis elegans* L1 arrest

**DOI:** 10.1093/g3journal/jkac092

**Published:** 2022-04-22

**Authors:** Jingxian Chen, Linda Y Tang, Maya E Powell, James M Jordan, L Ryan Baugh

**Affiliations:** 1 Department of Biology, Duke University, Durham, NC 27708, USA; 2 Center for Genomic and Computational Biology, Duke University, Durham, NC 27708, USA

**Keywords:** L1 diapause, L1 arrest, quiescence, starvation, PTEN, DAF-18

## Abstract

Mutations in the well-known tumor suppressor *PTEN* are observed in many cancers. PTEN is a dual-specificity phosphatase that harbors lipid and protein-phosphatase activities. The *Caenorhabditis elegans PTEN* ortholog is *daf-18*, which has pleiotropic effects on dauer formation, aging, starvation resistance, and development. Function of 3 *daf-18* point-mutants, *G174E*, *D137A*, and *C169S*, had previously been investigated using high-copy transgenes in a *daf-18* null background. These alleles were generated based on their mammalian counterparts and were treated as though they specifically disrupt lipid or protein-phosphatase activity, or both, respectively. Here, we investigated these alleles using genome editing of endogenous *daf-18*. We assayed 3 traits relevant to L1 starvation resistance, and we show that each point mutant is essentially as starvation-sensitive as a *daf-18* null mutant. Furthermore, we show that *G174E* and *D137A* do not complement each other, suggesting overlapping effects on lipid and protein-phosphatase activity. We also show that each allele has strong effects on nucleocytoplasmic localization of DAF-16/FoxO and dauer formation, both of which are regulated by PI3K signaling, similar to a *daf-18* null allele. In addition, each allele also disrupts M-cell quiescence during L1 starvation, though D137A has a weaker effect than the other alleles, including the null. Our results confirm that *daf-18/PTEN* is important for promoting starvation resistance and developmental arrest and that it is a potent regulator of PI3K signaling, and they highlight challenges of using genetic analysis to link specific DAF-18/PTEN enzymatic activities to particular phenotypes.

## Introduction 

Tumor suppressor Phosphatase and Tensin homolog (PTEN) is frequently mutated in many human cancers (Yin and Shen 2008; Lee *et al.* 2018). Abnormal PTEN activity is also implicated in neuronal disorders, autism, and aging (Redfern *et al.* 2010; Domanskyi *et al.* 2011; Tait *et al.* 2013; Johnston and Raines 2015; Knafo *et al.* 2016; Busch *et al.* 2019). PTEN is a dual-specificity phosphatase harboring lipid and protein-phosphatase activities. PTEN dephosphorylates phosphatidylinositol (3,4,5)-trisphosphate (PIP3) as a lipid phosphatase to counteract phosphoinositide 3-kinase (PI3K) signaling. Disruption of the lipid-phosphatase activity is associated with tumorigenesis and metabolic diseases like diabetes (Engelman *et al.* 2006). The protein-phosphatase activity contributes to PTEN’s tumor-suppressor function as well, regulating cell growth, proliferation, migration, and invasion (Tamura *et al.* 1998; Gu *et al*. 1999, 2011; Zhang *et al.* 2011).

Studies of PTEN function have employed *PTEN* missense mutants that selectively disrupt lipid or protein-phosphatase activities. Among these mutants, *PTEN(G129E)*, *PTEN(Y138L)*, and *PTEN(C124S)* are most widely used. *PTEN(G129E)* has little activity against PIP3 but retains a large percentage of the protein-phosphatase activity (Myers *et al.* 1997; Furnari *et al.* 1998; Myers *et al.* 1998; Ramaswamy *et al.* 1999; Han *et al.* 2000; Davidson *et al.* 2010; Rodríguez-Escudero *et al.* 2011); *PTEN(Y138L)* has barely any protein-phosphatase activity but retains most activity against PIP3 (Davidson *et al.* 2010); *PTEN(C124S)* eliminates nearly all lipid and protein-phosphatase activity (Myers *et al.* 1997, 1998; Ramaswamy *et al.* 1999; Davidson *et al.* 2010; Rodríguez-Escudero *et al.* 2011).

**Fig. 1.**
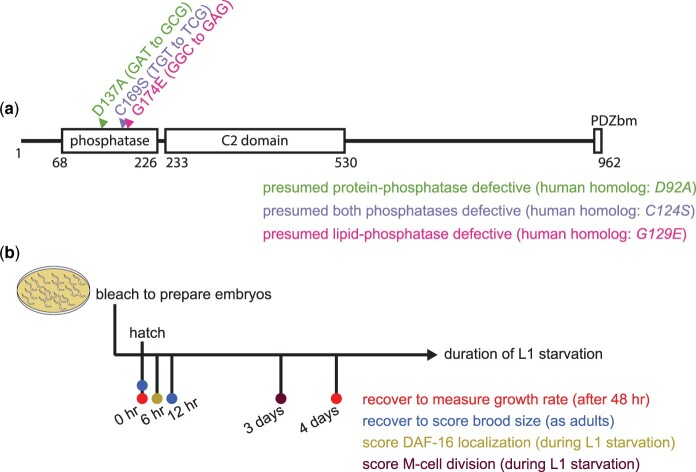
Schematic of DAF-18 protein and experimental design. a) DAF-18 full-length protein sequence was subjected to domain prediction using InterPro (Blum *et al.* 2021). A dual-specificity lipid/protein-phosphatase domain homologous to that of PTEN and a tensin-type C2 domain that can mediate Ca^2+^-independent membrane recruitment of proteins (Lee *et al.* 1999) are indicated. Three C-terminal amino acids (TKV) constitute a PDZ-binding motif (PDZbm) in PTEN (Kim *et al.* 1995; Songyang *et al.* 1997; Steck *et al.* 1997), but that motif is not conserved in DAF-18 (IYL), though DAF-18 can bind PDZ (An *et al.* 2019). The 3 *daf-18* missense alleles analyzed in this study are also indicated along with the homologous *PTEN* mutants. The same color-coding scheme for these alleles is used throughout the figures. b) Well-fed gravid adult worms of different genotypes were hypochlorite treated to obtain embryos, which were immediately placed in starvation cultures to hatch and enter L1 arrest (for experimental details, see Materials and methods). Time points of different assays performed are indicated with each color representing one assay.

The sole *Caenorhabditis* *elegans* PTEN ortholog DAF-18 shares significant sequence identity with its human counterpart in the phosphatase and C2 domains (Ogg and Ruvkun 1998; Mihaylova *et al.* 1999; Fig. 1a). *daf-18* regulates longevity, formation of dauer larvae in response to adverse conditions (high population density, low nutrient availability, and other forms of stress), and developmental arrest and survival of starved L1-stage larvae (L1 arrest), each of which is also regulated by PI3K signaling (Murphy and Hu 2013; Baugh and Hu 2020). *PTEN* transgenes can rescue *daf-18* dauer formation defective (Daf-d) and shortened-lifespan phenotypes (Solari *et al.* 2005), suggesting functional conservation. Given sequence and functional homology, researchers have fruitfully translated findings between *C. elegans* and mammals. In addition, research in *C. elegans* has taken advantage of *daf-18* mutations that correspond to the aforementioned *PTEN* mutations in an effort to connect specific enzymatic activities to specific phenotypes.*daf-18(G174E)* is the *C. elegans* homolog of *PTEN(G129E)*, and it is presumed to specifically disrupt lipid-phosphatase activity (Fig. 1a). Transgenic expression of *daf-18(G174E)* is unable to rescue the *daf-18* Daf-d, shortened-lifespan, and L1 arrest-defective (Q-cell division) phenotypes, but it can weakly rescue the vulval-induction phenotype (Solari *et al.* 2005; Nakdimon *et al.* 2012; Zheng *et al.* 2018b). These results suggest that lipid-phosphatase activity is important for DAF-18 to regulate dauer formation, aging, developmental arrest, and to a lesser extent vulval induction, and that protein-phosphatase activity may be required to inhibit vulval induction.

PTEN residue Y138 is not conserved in DAF-18, and there is no *daf-18* mutation homologous to *PTEN(Y138L)*. Nonetheless, *PTEN*(*D92A)*, which “traps” substrate but does not dephosphorylate it (Flint *et al.* 1997; Tamura *et al.* 1998), has a *C. elegans* homolog, *daf-18(D137A)* (Fig. 1a). By extension, D137A has been presumed to specifically disrupt DAF-18 protein-phosphatase activity (Zheng *et al.* 2018b). DAF-18(D137A) shows increased binding of its putative substrate VAB-1 and fails to decrease VAB-1 auto-phosphorylation to the level caused by wild-type DAF-18 (Brisbin *et al.* 2009). Additionally, transgenic expression of *daf-18(D137A)* was able to rescue the *daf-18* L1 arrest-defective (Q-cell division) phenotype, but *daf-18(G174E)* was not (Zheng *et al.* 2018b). Given different results with each allele, these results support the idea that *daf-18(D137A)* selectively disrupts protein-phosphatase activity while leaving lipid-phosphatase activity intact. They also suggest that DAF-18 regulates Q-cell division during L1 arrest independent of its protein-phosphatase activity. *daf-18(C169S)* corresponds to *PTEN(C124S)* (Fig. 1a) and is thought to affect both lipid and protein-phosphatase activities, since the mutated cysteine is in the catalytic core (Lee *et al.* 1999). Like DAF-18(D137A), DAF-18(C169S) also decreases VAB-1 auto-phosphorylation less than wild-type DAF-18 (Brisbin *et al.* 2009). Unlike *daf-18(D137A)*, transgenic expression of *daf-18(C169S)* fails to rescue the *daf-18* L1 arrest-defective (Q-cell division) phenotype (Zheng *et al.* 2018b), further suggesting that the lipid-phosphatase activity is essential to maintaining Q-cell quiescence during L1 arrest.

If *C. elegans* embryos hatch without food, they remain developmentally arrested in the first larval stage (L1 arrest or L1 diapause) (Baugh and Hu 2020). The transcription factor DAF-16/FoxO, which is inhibited by insulin/insulin-like growth factor (IGF) and AGE-1/PI3K signaling (Lin *et al.* 1997; Ogg *et al.* 1997), is a critical regulator of L1 arrest (Baugh and Sternberg 2006). AGE-1/PI3K signaling transduces signals from activated DAF-2/IGF receptor (IGFR) and is opposed by DAF-18/PTEN in that the conversion of PIP2 into PIP3 by AGE-1 is reversed by DAF-18 via its lipid-phosphatase activity (Morris *et al.* 1996; Ogg and Ruvkun 1998; Mihaylova *et al.* 1999). As a result, null mutants of *daf-16* and *daf-18* are starvation-sensitive and L1 arrest-defective (Baugh and Sternberg 2006; Fukuyama *et al.* 2006; 2012; Cui *et al.* 2013; Kaplan *et al.* 2015; Zheng *et al.* 2018a, 2018b; Fry *et al.* 2021).

Here, we present phenotypic analysis of *daf-18(G174E)*, *daf-18(D137A)*, and *daf-18(C169S)* in the context of L1 arrest, with focus on starvation resistance. Our initial objective was to identify which DAF-18 enzymatic activities are required for starvation resistance, but we were also concerned about the specificity of each allele with respect to enzymatic activity. Rather than relying on transgenes, we used CRISPR-Cas9 genome editing to modify the endogenous *daf-18* locus to produce *daf-18(G174E)*, *daf-18(D137A)*, and *daf-18(C169S)* mutants. We used 3 complementary assays for L1 starvation resistance: L1 starvation survival, growth during recovery from L1 starvation, and fecundity following L1 starvation (Fig. 1b). We also assayed M-cell divisions and DAF-16::GFP nucleocytoplasmic localization during L1 arrest as well as high-temperature dauer formation with *daf-2/IGFR* RNAi. All 3 alleles behaved similar to a *daf-18* null mutant in each assay with the exception of the M-cell division assay. To address potential overlapping effects of each mutation on DAF-18 enzymatic activity, we assayed starvation resistance of *trans*-heterozygotes of *D137A* and *G174E*, and these alleles did not complement each other. This result suggests that at least one of the 2 alleles appreciably disrupts both enzymatic activities, confounding the ability to infer causal relationships between a specific enzymatic activity and a specific phenotype. Similar results and conclusions were drawn in a study by Wittes and Greenwald (submitted in parallel) examining somatic gonad and germ-cell quiescence in dauer larvae. Collectively, these 2 studies suggest the need for caution in interpreting results of genetic analysis using these missense alleles.

## Materials and methods

### Strains used in this study

Wild-type strain N2 is from the Sternberg Lab collection.

PD4666 *ayIs6[hlh-8p::GFP + dpy-20(+)] X* was obtained from the CGC.

IC166 *daf18(ok480) IV* was obtained from the Chin-Sang Lab at Queen’s University.

OH16024 *daf-16(ot971[daf-16::GFP]) I* was obtained from the Hobert Lab at Columbia University.

### Strains generated in this study

PHX1516 *daf-18(syb1516) IV*, PHX1615 *daf-18(syb1615) IV*, and PHX1618 *daf-18(syb1618) IV* were generated by SunyBiotech and have CRISPR-Cas9 engineered endogenous alleles *C169S*, *D137A*, and *G174E*, respectively.

LRB434: *syb1615* backcrossed 4 times to SunyBiotech N2

LRB435: *syb1618* backcrossed 4 times to SunyBiotech N2

LRB436: *syb1516* backcrossed 4 times to SunyBiotech N2

LRB455: *daf-16(ot971[daf-16::GFP]) I; daf-18(ok480) IV*

LRB465: *daf-16(ot971[daf-16::GFP]) I; daf-18(syb1618) IV*

LRB467: *daf-16(ot971[daf-16::GFP]) I; daf-18(syb1615) IV*

LRB468: *daf-16(ot971[daf-16::GFP]) I; daf-18(syb1516) IV*

LRB472: *daf-18(syb1615) IV; ayIs6[hlh-8p::GFP + dpy-20(+)] X*

LRB477: *daf-18(ok480) IV; ayIs6[hlh-8p::GFP + dpy-20(+)] X*

LRB478: *daf-18(syb1516) IV; ayIs6[hlh-8p::GFP + dpy-20(+)] X*

LRB479: *daf-18(syb1618) IV; ayIs6[hlh-8p::GFP + dpy-20(+)] X*

### Starvation survival

All strains assayed in this study were maintained on *Escherichia* *coli* OP50 nematode growth medium (NGM) plates and were well-fed for at least 3 generations before being used in experiments. Worms were grown and starved at 20^°^C. Gravid adult worms were hypochlorite treated (“bleached”) to collect embryos, which were then resuspended, washed, and cultured in S-basal (including 0.1% EtOH). Cultures had ∼1 embryo/µl in 5 ml and were placed in 16 mm glass tubes on a tissue-culture roller drum at 20°C so they hatch and enter L1 arrest (Hibshman *et al.* 2021). The day after hypochlorite treatment, and again every day after that, a 100 µl aliquot of each starvation culture was plated on a 6 cm NGM plate around an *E. coli* OP50 lawn in the center. The number of larvae in the aliquot was recorded (total plated). Two days later, the number of live worms on the lawn was recorded (total alive). Proportion alive was determined as total alive divided by total plated.

### Growth rate measurement

Strains were treated as described above in “Starvation survival,” and the same worm population was used both here and in the survival assay. Worms were grown at 20^°^C. Five hundred microliters of starvation culture was plated on 10 cm NGM plates seeded with *E. coli* OP50 to support larval development (recovery plates). For the control condition (no starvation), embryos were plated directly after hypochlorite treatment, before starvation cultures were placed on the roller drum. For the starved condition, 500 µl of the starvation culture was plated 4 days after hypochlorite treatment. Worms were washed off plates with S-basal and transferred to unseeded (no OP50) 10 cm NGM plates for imaging using ZEISS SteREO Discovery.V20. Because L1 larvae take ∼12 h to hatch following hypochlorite treatment, control and starved worms were imaged 60 and 48 h after plating, respectively. This approach enabled measurements of body length after ∼48 h of feeding for both populations. The image-analysis program WormSizer was used to determine worm length (Moore *et al.* 2013).

### Total brood size measurement

Strains were treated as described above, and the same worm population was used as for growth rate measurement and for starvation survival. Worms were grown at 20°C. For the control condition (no starvation), 18 worms were singled out per genotype from the control recovery plate of the growth-rate assay onto 6 cm NGM plates seeded with OP50 the day after hypochlorite treatment. For the starved condition, a 50 µl aliquot of the starvation culture was plated on a 6 cm NGM plate with OP50 24 h after hypochlorite treatment (∼12-h starvation) for each genotype, and 18 worms were singled out onto 6 cm NGM plates with OP50 the next day. Starting the day after singling out, and every day after for 5, each worm was transferred to a new 6 cm NGM plate with OP50. The total brood size of each worm was determined from the number of progeny on each plate. Worms that died during the process, including due to internal hatching (“bagging”), were recorded, and their total brood sizes were included in the reported results since gonad abnormalities and bagging are consequences of L1 starvation (Jordan *et al.* 2019).

### M-cell division

Strains were treated as described above in “Starvation survival.” Three days after hypochlorite treatment, larvae were mounted on 4% Noble agar pads on microscope slides and covered by a glass cover slip to be examined for M-cell division at 200x total magnification on a Zeiss Axio Imager A1. 100 L1 larvae were scored for each condition in each replicate.

### DAF-16::GFP localization

Strains were treated as described above in “Starvation survival.” Eighteen hours after hypochlorite treatment (∼6 h of starvation), a 700 µl aliquot of the starvation culture was centrifuged in a 1.7 ml Eppendorf tube at 3,000 rpm for 30 s to pellet L1 larvae. 1.5 µl of worm pellet was pipetted into the center of a slide with a 4% Noble agar pad, and a glass cover slip was immediately placed on top. A timer was set for 3 min, and the slide was systematically scanned with each individual worm scored for nucleocytoplasmic localization specifically within intestinal cells at 1000x on a Zeiss Axio Imager A1. At least 50 larvae were scored per replicate. Nucleocytoplasmic localization of DAF-16::GFP was scored in intestinal cells and assigned to one of 4 categories: nuclear, more nuclear, more cytoplasmic, and cytoplasmic. “More nuclear” and “more cytoplasmic” are intermediate categories between nuclear and cytoplasmic, with localization being more nuclear or cytoplasmic, respectively. Scoring for each slide stopped after 3 min in order to avoid confounding effects of the worms being mounted on slides.

### Dauer-formation assay

Single *E. coli* HT115 colonies carrying pAD12 for empty vector (EV) or pAD48 for *daf-2* RNAi grown on 12.5 µg/ml tetracycline and 100 µg/ml carbenicillin LB plates at 37°C were inoculated into LB starter cultures with the same concentration of tetracycline and half the concentration of carbenicillin. Starter cultures were incubated overnight at 37°C with shaking and then used to inoculate larger cultures of terrific broth (TB) with 25 µg/ml carbenicillin which were incubated overnight at 37°C with shaking. Cells from large cultures were spun down and resuspended at a final density of 250 mg/ml in S-complete with 15% glycerol. Aliquots were frozen and thawed only once and seeded onto 6 cm NGM plates containing 25 µg/ml carbenicillin and 1 mM IPTG. Lawns were allowed to grow overnight at room temperature before plating worms. For each genotype and each replicate, 7 L4 worms were picked onto EV or *daf-2* RNAi plates and grown at 27°C. Twenty hours after plating, they were removed but their progeny were allowed to continue growing at 27°C. Forty-eight hours later, the total number of worms and the number of dauers were recorded for each plate and the proportion of dauers was calculated. Dauers were identified by visual inspection on plates (relatively long, slender shape), and worms that had bypassed dauer formation had vulvas and were typically adults.

## Results

### *daf-18(G174E)*, *daf-18(D137A)*, and *daf-18(C169S)* are starvation-sensitive

We used CRISPR-Cas9 to edit the laboratory reference N2 genome, producing *daf-18(G174E)*, *daf-18(D137A)*, and *daf-18(C169S)* mutants (Fig. 1a). We backcrossed these mutants 4 times, and we assayed L1 starvation resistance along with wild type and a null allele of *daf-18* (*ok480*). The ability to survive starvation is clearly an indication of starvation resistance, but L1 starvation also affects growth and reproductive success after recovery, and there are genotypes and conditions that affect growth and fecundity following starvation without appreciably affecting starvation survival (Baugh and Hu 2020). We therefore assayed starvation survival as well as growth and total brood size after recovery (Fig. 1b). Because DAF-18 promotes DAF-16 activity via its well-established antagonism toward PI3K signaling, and *daf-16* mutants are starvation-sensitive, *daf-18(G174E)* (presumed lipid-phosphatase defective) and *daf-18(C169S)* (presumed both phosphatase-activities defective) were expected to be starvation-sensitive. In contrast, it was unclear whether *daf-18(D137A)* (presumed protein-phosphatase defective) would affect starvation resistance.

**Fig. 2.**
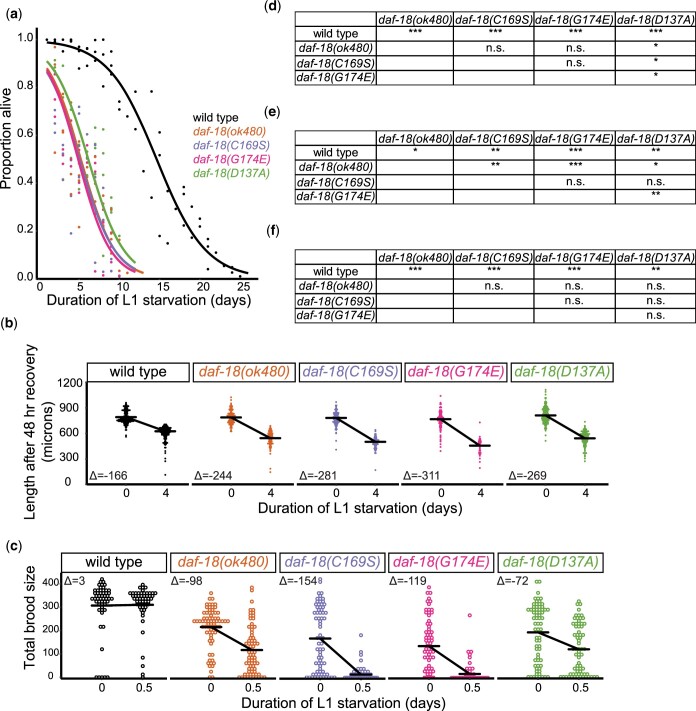
*daf-18* mutants are sensitive to L1 starvation. a and d) Survival was scored daily during L1 arrest, and survival curves were fit using logistic regression. Four biological replicates were performed. For each genotype and each replicate, quasi-binomial logistic regression was performed with the response variable being proportion alive and the explanatory variable being duration of L1 starvation. Half-lives (time corresponding to when the proportion alive is half of the starting value) were estimated by regression and were subjected to Bartlett’s test, which indicated homogeneity of variance across all genotypes. Two-tailed unpaired *t*-tests with variance pooled across genotypes were performed on half-lives to calculate *P*-values for comparisons between genotypes. b and e) Body length after 48-h recovery on 10 cm NGM plates with *E. coli* OP50 for food was measured using the program WormSizer for image analysis (Moore *et al.* 2013) for starved (4-day L1 starvation) and control (no starvation) conditions. Four biological replicates were performed. C and F) Total brood size was scored for each worm in starved (12-h L1 starvation) and control (no starvation) conditions. Three biological replicates were performed. b, c, e, and f) A linear mixed effects (lme) model was fit to the data with the response variable being body length or brood size, fixed effects being the interaction between starvation and genotype, and the random effect being replicates. *P*-values for interaction terms were extracted. Horizontal bars represent estimates calculated by lme models. Δ values are differences between estimates in starved and control conditions. Each dot represents an observation with a single worm. d–f) *P < 0.05; **P < 0.01; ***P < 0.001; n.s. not significant.

We found that all 3 missense alleles of *daf-18* are very sensitive to L1 starvation. All 3 mutants and the null mutant survived L1 starvation for a significantly shorter time than wild type (Fig. 2, a and d). Likewise, all 4 *daf-18* mutants displayed significantly greater decreases in growth after 4 days of L1 starvation compared to wild type (Fig. 2, b and e). Furthermore, all 4 mutants showed significant reduction in fecundity after 12 h of L1 starvation (Fig. 2, c and f). This last result demonstrates a striking cost of starvation on reproductive success in *daf-18* mutants, given little to no effect of this brief 12-h starvation on the wild-type control. Together, these results demonstrate that *daf-18(G174E)*, *daf-18(D137A)*, and *daf-18(C169S)* are each very sensitive to L1 starvation (based on 3 different assays) comparable to a null allele.

### M cells divide in *daf-18(G174E)*, *daf-18(D137A)*, and *daf-18(C169S)* during L1 arrest

In addition to adapting to survive and recover from L1 starvation, wild-type worms remain in a developmentally arrested state while starved. There is a single M mesoblast cell at hatching, and it divides approximately halfway through the first larval stage in fed worms and goes on to produce 18 descendants by the L1 molt (Sulston and Horvitz 1977). However, the M cell does not divide during L1 starvation in wild-type worms (Baugh and Sternberg 2006; Kaplan *et al.* 2015). Developmental arrest, like starvation resistance, is affected by *daf-18*, so we assayed M-cell divisions in starved L1 larvae. We scored the number of M cells using an *hlh-8p*::GFP reporter (Harfe *et al.* 1998) in *daf-18(G174E)*, *daf-18(D137A)*, and *daf-18(C169S)*, as well as *daf-18(ok480)* and wild type, after 3 days of starvation (Fig. 1b). M-cell quiescence requires *daf-18* and *daf-16*, and so, by the same reasoning as for starvation resistance, we expected *daf-18(G174E)* (presumed lipid-phosphatase defective) and *daf-18(C169S)* (presumed both phosphatase-activities defective) to have M-cell divisions, but it was unclear whether *daf-18(D137A)* (presumed protein-phosphatase defective) would affect M-cell arrest.

**Fig. 3.**
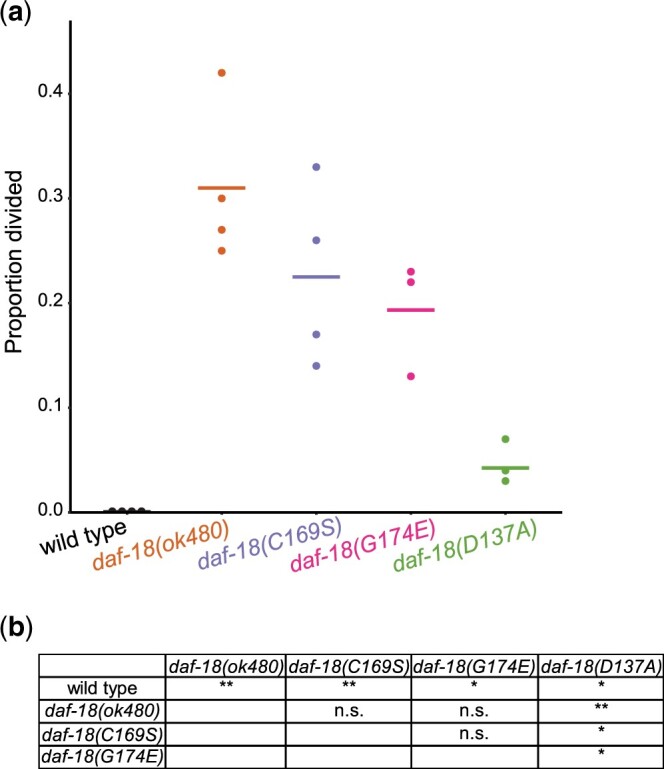
*daf-18* mutants have M-cell divisions during L1 starvation. a) Proportion of starved L1 larvae with more than one M cell after 3 days of L1 arrest. Three biological replicates were performed. One hundred larvae were scored per genotype per replicate. Horizontal bars represent average proportions. b) Bartlett’s test was performed on proportion divided, which indicated heterogeneity of variance across all genotypes. One-tailed unpaired *t*-tests (without pooling variance across genotypes) were conducted to compare *daf-18* mutants to wild type. Two-tailed unpaired *t*-tests (without pooling variance across genotypes) were conducted to compare between *daf-18* mutants. *P < 0.05; **P < 0.01; n.s. not significant.

We found that all 3 missense alleles and the null allele of *daf-18* display M-cell divisions during L1 starvation (Fig. 3, a and b). However, unlike starvation resistance, *daf-18(D137A)* (presumed protein-phosphatase defective) was clearly different than the other alleles, with significantly fewer animals with a divided M cell, though still significantly more than wild type (zero). These results show that each of the *daf-18* missense mutations confers an L1 arrest-defective phenotype, but they also reveal a difference in penetrance among the alleles.

### DAF-16/FoxO localizes to the cytoplasm during starvation in *daf-18(G174E)*, *daf-18(D137A)*, and *daf-18(C169S)*

Though it is possible that DAF-18 regulates L1 starvation resistance and developmental arrest through its putative protein-phosphatase activity, we grew concerned about the specificity with which the *D137A* and *G174E* alleles disrupt protein and lipid-phosphatase activities, respectively. We decided to look at a direct target of AGE-1/PI3K signaling, DAF-16/FoxO, at the subcellular level. When insulin/IGF signaling is low, such as during starvation, DAF-16 localizes to the nucleus to regulate transcription, but when insulin/IGF signaling, and thus AGE-1/PI3K signaling, is activated, DAF-16 localizes to the cytoplasm where it is inactive (Henderson and Johnson 2001; Lee *et al.* 2001; Lin *et al.* 2001). We expected disruption of DAF-18 lipid-phosphatase activity to render DAF-16 more cytoplasmic during starvation as a result of increased AGE-1/PI3K signaling, but we reasoned that it is much less likely that disruption of its protein-phosphatase activity would affect localization.

**Fig. 4.**
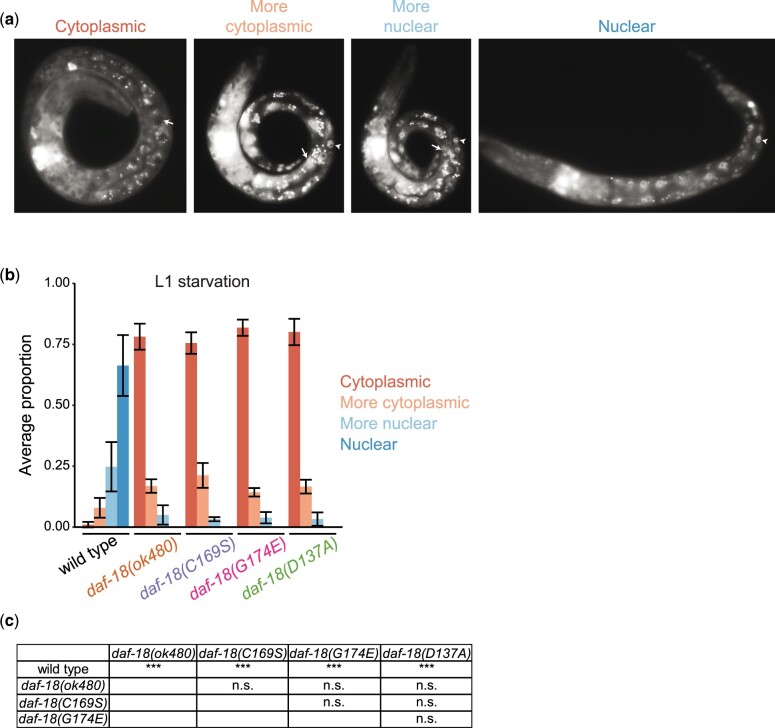
DAF-16 localizes to the cytoplasm during L1 starvation in *daf-18* mutants. a) Representative images of each of 4 categories of DAF-16::GFP nucleocytoplasmic localization in intestinal cells taken at 1,000x. Additional cells are also visible in the images but were not evaluated during scoring. Arrows and arrowheads annotate representative cells with cytoplasmic and nuclear DAF-16::GFP localization, respectively. b) Average proportion of larvae with cytoplasmic, more cytoplasmic, more nuclear, and nuclear DAF-16::GFP localization in intestinal cells after 6 h of L1 starvation. Four biological replicates were performed. At least 50 larvae were scored per genotype per replicate. Error bars represent standard deviations. c) Cochran-Mantel-Haenszel chi-squared tests were conducted for each pair of comparisons between genotypes. ***P < 0.001; n.s. not significant.

We scored DAF-16 subcellular localization after 6 h of L1 starvation (Figs. 1b and 4a). The vast majority of wild-type larvae had DAF-16::GFP localized to the nucleus (Fig. 4, b and c), as expected. In contrast, the vast majority of larvae had DAF-16::GFP localized to the cytoplasm in *daf-18(G174E)*, *daf-18(D137A)*, *daf-18(C169S)*, and *daf-18(ok480)*. These results demonstrate that all 3 missense alleles as well as the null allele have a strong effect on DAF-16::GFP localization. It is a formal possibility that DAF-18 regulates DAF-16 localization independent of AGE-1/PI3K signaling through its protein-phosphatase activity, but we believe the simplest interpretation of these results is that the *D137A* allele (presumed protein-phosphatase defective) lacks specificity and also disrupts the lipid-phosphatase activity of DAF-18.

**Fig. 5.**
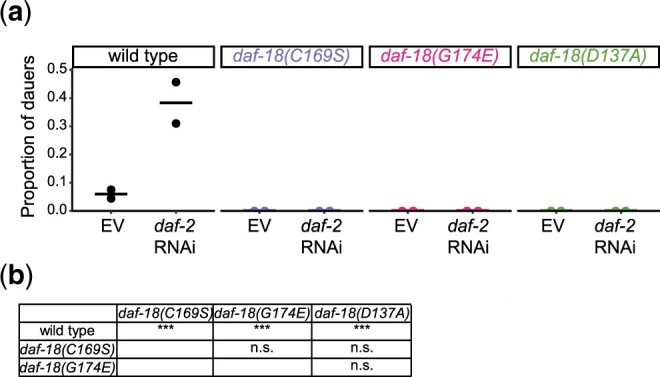
*daf-18* mutants are dauer-formation defective. a) Proportion of dauers among worms grown at 27°C on EV (negative control) or *daf-2* RNAi food. Two biological replicates were performed. At least 100 worms were scored per genotype per RNAi treatment per replicate. Horizontal bars represent average proportions. b) A two-way analysis of variance (ANOVA) test and *post hoc* Tukey tests were conducted to compare between genotypes. ***P < 0.001; n.s. not significant.

### *daf-18(G174E)*, *daf-18(D137A)*, and *daf-18(C169S)* are dauer-formation defective

Insulin/IGF and AGE/PI3K signaling is also a critical regulator of dauer formation (Hu 2007; Murphy and Hu 2013), and the dauer formation constitutive (Daf-c) phenotype of *daf-2/IGFR* mutants is suppressed by disruption of *daf-18* (Mihaylova *et al.* 1999). We reasoned that in dauer-forming conditions provided by high temperature (27°C) and *daf-2/IGFR* RNAi, disruption of DAF-18 lipid-phosphatase but not protein-phosphatase activity would rescue DAF-2/IGFR signaling and reduce dauer formation. We found that less than 10% of wild-type worms grown at high temperature without RNAi (control conditions) developed into dauer larvae, and that over 30% of worms grown at high temperature with *daf-2* RNAi (dauer-forming conditions) became dauers, as expected. In contrast, no *daf-18(G174E)*, *daf-18(D137A)*, or *daf-18(C169S)* worms became dauers in either control or dauer-forming conditions (Fig. 5). These results demonstrate that all 3 *daf-18* missense alleles suppress dauer formation in response to reduced insulin/IGF signaling and high temperature. Notably, Wittes and Greenwald (submitted in parallel) also report that *daf-18(D137A)* (presumed protein-phosphatase defective) is defective at dauer development while focusing on somatic gonad development and germ cell proliferation in dauer larvae induced by *daf-7/TGF-β* mutation. As for the other phenotypes we examined, it is possible that DAF-18 regulates dauer formation through an unknown mechanism involving its protein-phosphatase activity, but these results along with those of Wittes and Greenwald (submitted in parallel) challenge the notion that *D137A* disrupts DAF-18 protein-phosphatase activity without appreciable effects on its lipid-phosphatase activity.

### *daf-18(D137A)* and *daf-18(G174E)* do not complement each other

**Fig. 6.**
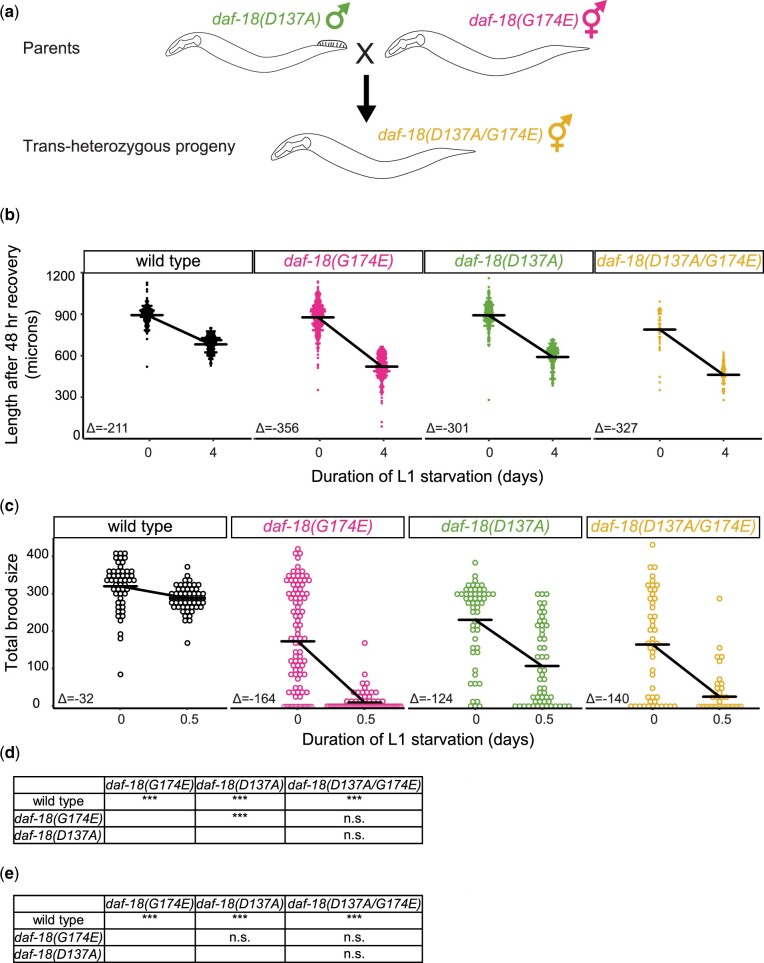
*daf-18(D137A)* and *daf-18(G174E)* do not complement each other. a) Cross scheme for generating *trans*-heterozygotes of *daf-18 D137A* and *G174E* alleles. b,d) Body length after 48-h recovery on food was measured using the program WormSizer for image analysis (Moore *et al.* 2013) for starved (4-day L1 starvation) and control (no starvation) conditions. Body lengths of both males and hermaphrodites were recorded, and no significant difference was found between them, but only the results from hermaphrodites are reported. c,e) Total brood size was scored for each worm in starved (12-h L1 starvation) and control (no starvation) conditions. b–e) Three biological replicates were performed. Mating efficiency was carefully checked by recording numbers of males and hermaphrodites in the images used for WormSizer and only replicates with a ∼1:1 male to hermaphrodite ratio were included (brood size was determined from the same worm populations). A linear mixed effects (lme) model was fit to the data with the response variable being body length or brood size, fixed effects being the interaction between starvation and genotype, and the random effect being replicates. *P*-values for interaction terms are reported. Horizontal bars represent estimates calculated by lme models. Δ values are differences between estimates in starved and control conditions. Each dot represents an observation with a single worm. d, e) ***P < 0.001; n.s. not significant.

The results presented thus far suggest that either DAF-18 affects a variety of phenotypes through its protein-phosphatase activity in addition to its better-characterized lipid-phosphatase activity, or that *daf-18(D137A)* actually disrupts the lipid-phosphatase activity rather than being selective for the protein-phosphatase activity. We reasoned that by analyzing *D137A/G174E trans*-heterozygotes we could assess whether these 2 alleles have overlapping effects on DAF-18 function or if they are actually specific to each enzymatic activity. That is, if these alleles are truly specific, then they should complement each other and *trans*-heterozygotes should be wild-type-like. We produced *daf-18(D137A/G174E) trans*-heterozygotes (Fig. 6a), and we assayed their effect on L1 starvation resistance (Fig. 1b) along with homozygous mutants and wild type. We chose starvation resistance since this is our primary focus and we have the most data on this trait. However, our starvation survival and growth assays require large numbers of animals, and we were not able to obtain enough cross-progeny (*trans*-heterozygotes) for both assays. We found that growth of *daf-18(D137A/G174E)* animals was more impaired after 4 days of starvation than wild-type (Fig. 6, b and d), suggesting starvation sensitivity. Likewise, *daf-18(D137A/G174E)* animals produced fewer progeny than wild type after 12 h of starvation (Fig. 6, c and e). Not only were the *trans*-heterozygotes significantly different from wild type in both assays, but they were statistically indistinguishable from each homozygous mutant in each assay. These results clearly demonstrate that *D137A* and *G174E* do not complement each other, suggesting overlapping effects on DAF-18 function.

## Discussion

We used genome editing to produce *daf-18(G174E)*, *daf-18(D137A)*, and *daf-18(C169S)* alleles, and we determined their effects on starvation resistance and developmental quiescence during *C. elegans* L1 arrest. We hoped to use these missense alleles to dissect the enzymatic activities of DAF-18/PTEN, delineating the function of its lipid and protein-phosphatase activities with respect to some of its mutant phenotypes. We found that all 3 alleles reduced starvation resistance, eliminated nuclear localization of DAF-16::GFP during starvation, and suppressed dauer formation. In each case, penetrance was comparable to a null allele (except a null allele was not included in the dauer-formation assay). Each missense allele also caused an L1 arrest-defective phenotype (M-cell divisions), though penetrance varied, with *D137A* (presumed protein-phosphatase defective) displaying significantly lower penetrance. There is ample evidence that DAF-18 affects each of these phenotypes through its lipid-phosphatase activity, but there is no evidence that its protein-phosphatase activity is involved, though it is possible that DAF-18 dephosphorylates a protein in the AGE-1/PI3K pathway (Shi *et al.* 2014). We therefore analyzed *trans*-heterozygotes of *D137A* and *G174E*, and these mutants failed to complement each other's starvation-sensitive phenotypes. Together these results suggest that D137A (presumed protein-phosphatase defective) and possibly G174E (presumed lipid-phosphatase defective) disrupt more than one of these 2 DAF-18 activities. Either allele could also affect DAF-18 expression levels, but we cannot distinguish between these possibilities with genetic analysis alone.

Wittes and Greenwald (submitted in parallel) analyzed dauer development in *D137A/G174E trans-*heterozygotes and also observed lack of complementation. They also showed that *daf-18* is not haplo-insufficient. These results further suggest overlapping effects of each allele on DAF-18 function, and they also concluded that these alleles do not specifically disrupt one or the other enzymatic activity of DAF-18.

In vitro and in vivo phosphatase assays performed with PTEN greatly outnumber those performed with DAF-18. In vitro experiments actually suggest that PTEN(D92A) has disrupted lipid-phosphatase activity (Lee *et al.* 1999; Xiao *et al.* 2007; Rodríguez-Escudero *et al.* 2011) in addition to loss of protein-phosphatase activity (Tamura *et al.* 1999). In addition, in vitro and in vivo studies suggest that although PTEN(G129E) retains little lipid-phosphatase activity (Myers *et al.* 1998; Rodríguez-Escudero *et al.* 2011), it also lacks some protein-phosphatase activity (Furnari *et al.* 1998; Myers *et al.* 1998; Ramaswamy *et al.* 1999). The crystal structure of PTEN reveals a sole active site pocket that presumably has catalytic activity toward PIP3 and phosphoproteins (Lee *et al.* 1999), consistent with each mutation affecting both activities. Moreover, although DAF-18 and PTEN share the same PTP signature motif in the catalytic core, they have less homology in the WPD loop (88–98 in PTEN) and TI loop (160–171 in PTEN) that together with the P loop constitute the sidewalls of the active site pocket (Ogg and Ruvkun 1998; Lee *et al.* 1999; Mihaylova *et al.* 1999). Therefore, we believe extrapolation from the properties of these *PTEN* alleles to the homologous *daf-18* alleles should be made very cautiously.

In contrast to the other phenotypes assayed, *daf-18(D137A)* had significantly less effect on M-cell divisions during L1 starvation than the other alleles examined. Likewise, a high-copy *daf-18(D137A)* transgene functioned like wild-type *daf-18* when used to rescue Q-cell quiescence during L1 starvation in the null *daf-18(ok480)* background (Zheng *et al.* 2018b)*.* In contrast, *daf-18(C169S)* and *daf-18(G174E)* transgenes did not rescue quiescence. These results suggest that the effects of D137A and G174E on DAF-18 function do not completely overlap, as if there is a degree of specificity that can distinguish them in some phenotypic assays (consistent with biochemical characterization of their mammalian homologs; see above). The fact that *daf-18(D137A)* has less of a relative effect on M or Q-cell divisions than starvation resistance, DAF-16::GFP localization, or dauer development could reflect a difference in the relative sensitivity of the underlying biological process to the lipid and/or protein-phosphatase activities of DAF-18. However, our work together with that of Wittes and Greenwald (submitted in parallel) cautions against using missense alleles (D137A and G174E in particular) in an effort to causally connect specific DAF-18 enzymatic activities with specific phenotypes.

## Data availability

Data generated during the process of this study are available in this article. Strains are available upon request. Code for performing statistical tests with starvation survival data is available at https://github.com/jc271828/Chen2022 [accessed 2022 Apr 25].
